# Coordinated Binding of Single-Stranded and Double-Stranded DNA by UvsX Recombinase

**DOI:** 10.1371/journal.pone.0066654

**Published:** 2013-06-18

**Authors:** Robyn L. Maher, Scott W. Morrical

**Affiliations:** Department of Biochemistry, University of Vermont College of Medicine, Burlington, Vermont, United States of America; Saint Louis University, United States of America

## Abstract

Homologous recombination is important for the error-free repair of DNA double-strand breaks and for replication fork restart. Recombinases of the RecA/Rad51 family perform the central catalytic role in this process. UvsX recombinase is the RecA/Rad51 ortholog of bacteriophage T4. UvsX and other recombinases form presynaptic filaments on ssDNA that are activated to search for homology in dsDNA and to perform DNA strand exchange. To effectively initiate recombination, UvsX must find and bind to ssDNA within an excess of dsDNA. Here we examine the binding of UvsX to ssDNA and dsDNA in the presence and absence of nucleotide cofactor, ATP. We also examine how the binding of one DNA substrate is affected by simultaneous binding of the other to determine how UvsX might selectively assemble on ssDNA. We show that the two DNA binding sites of UvsX are regulated by the nucleotide cofactor ATP and are coordinated with each other such that in the presence of ssDNA, dsDNA binding is significantly reduced and correlated with its homology to the ssDNA bound to the enzyme. UvsX has high affinity for dsDNA in the absence of ssDNA, which may allow for sequestration of the enzyme in an inactive form prior to ssDNA generation.

## Introduction

Unrepaired DNA double-strand breaks are associated with increased risk of certain cancers in humans [1], [2]. Homologous recombination (HR) is used by cells for error-free repair of DNA double-strand breaks, and to restart stalled replication forks [3], [4]. Recombinases of the RecA/Rad51 family perform the central catalytic role in homologous recombination of DNA. Recombinases bind cooperatively to regions of single-stranded DNA (ssDNA) to form a presynaptic filament. The presynaptic filament is then aligned with homologous regions of double-stranded DNA (dsDNA), and a strand exchange occurs in which the single-stranded DNA invades and disrupts the double-stranded DNA to exchange out one of the strands. The recombinase filament then must dissociate from the DNA to allow for completion of the repair process [5], [6].

Presynaptic filament formation on ssDNA is necessary to activate the recombination activities of UvsX and other recombinases. Recombinases must also negotiate interactions with dsDNA during at least three stages of the strand exchange process: First, presynaptic filament assembly on ssDNA must occur in the presence of a large excess of dsDNA. Therefore recombinase filaments that form inappropriately on dsDNA must be resolved and the recombinase subunits redirected into productive filament assembly on ssDNA. Second, once presynaptic filaments assemble, they must recognize homologous dsDNA as a substrate. Third, following strand invasion, the recombinase filament bound to the double-stranded, heteroduplex product must dissociate to provide access to downstream DNA replication and repair machineries. Thus, recombinase-dsDNA interactions occur during presynapsis, synapsis, and postsynapsis stages of DNA strand exchange. Proper coordination between recombinase-ssDNA and dsDNA-binding activities must occur to ensure that recombination happens in a timely and efficient manner.

To effectively catalyze the strand exchange reaction, recombinases work in concert with several other proteins. These include single-stranded DNA binding proteins (SSBs) that denature secondary structure in single-stranded DNA and regulate access to ssDNA during various stages of DNA metabolism [7]. Most recombinases cannot compete with SSBs to gain access to ssDNA. As a result, recombinases require another family of proteins called recombination mediator proteins [8]. These proteins facilitate the displacement of SSBs and regulate the activity of recombinases such that recombination does not occur at inappropriate times or places in the genome. Evidence suggests that recombination mediator proteins also play important roles in directing recombinase assembly onto ssDNA during presynapsis [6], [9], [10]. However recombinases themselves must have some intrinsic properties that allow them to identify appropriate DNA substrates throughout the strand exchange process. It is these properties that are the subject of this study.

The general mechanisms of recombination described above are conserved throughout most DNA based organisms. The bacteriophage T4 recombination system is one of the most simple and robust [11], [12]. In comparison to the prokaryotic and eukaryotic systems, the T4 recombination system has relatively few regulatory and accessory factors. This makes UvsX ideal for detailed studies of the fundamental mechanism of recombinase-catalyzed DNA strand exchange, which is likely to be conserved in higher organisms. UvsX is orthologous to the bacterial RecA, the eukaryotic Rad51 and the archaeal RadA recombinase families [13]–[15].

The ssDNA binding properties of UvsX protein have been studied under a variety of conditions [16], [17]. UvsX binds cooperatively to long ssDNA molecules with a binding site size of 4 nucleotides per UvsX monomer. In the presence of ATP, DNA within the UvsX filament is stretched and underwound, disrupting base stacking interactions [18], [19]. UvsX/ssDNA filaments are stabilized in the stretched/underwound form by adenosine-5′-(3-thio)-triphosphate (ATPγS), a slowly hydrolyzed ATP analogue [16], [20]. Much less is known about the dsDNA binding properties of UvsX protein. It is known that UvsX binds to dsDNA with affinity that is at least as strong as its affinity for ssDNA [21] (H. Xu and S. Morrical, unpublished results). Unlike ssDNA, dsDNA binding does not activate the ATPase activity of UvsX [22]. Quantitative details of UvsX-dsDNA binding, and its relationship to nucleotide and ssDNA binding, are generally lacking, however.

To understand the mechanism by which UvsX discriminates between ssDNA and dsDNA, it is necessary to devise methods for measuring its affinity for one in the presence of the other. In this study we quantitatively characterize and directly compare UvsX interactions with short, fluorescently tagged homopolymeric ssDNA and dsDNA substrates. The use of these substrates eliminates secondary structure and long-range cooperative effects, revealing the fundamental allosteric effects caused by one DNA ligand on the binding of the other. We find here, as in other studies [21], that UvsX binds to dsDNA alone better than to ssDNA alone. We show that the two DNA binding sites of UvsX are regulated by the nucleotide cofactor ATP and are coordinated with each other such that in the presence of ssDNA, dsDNA binding affinity is reduced and correlated with its homology to the bound ssDNA. The data suggest a mechanism in which ssDNA binding allosterically reduces UvsX-dsDNA binding affinity, allowing UvsX to sample dsDNA for homology without the overabundance of heterologous dsDNA in the cell becoming inhibitory.

## Materials and Methods

### DNA Oligonucleotides and Nucleotide Cofactors

Oligonucleotide 1 (oligo 1) is a 25 mer of the sequence 5′-dA_22_XA_2_-3′ in which X is amino-modifier C2 dT (Glenn Research) and was synthesized and purified by HPLC by Biosynthesis Inc., Lewisville TX. The amino linker was reacted with AlexaFluor 546 carboxylic acid succinimidyl ester (Invitrogen, Eugene, OR) according to the supplier’s protocol to generate the labeled substrate. Spectral analysis indicated that the oligonucleotide was >95% labeled and free of non-covalently associated fluorophore. Oligonucleotide 2 (oilgo 2) is a 25 mer of the sequence dT_25_. Oligonucleotide 3 (oligo 3) is a 25 mer of the sequence dC_25_. Oligonucleotide 4 (oligo 4) is a 25 mer of the sequence dA_25_. Oligonucleotides 2–4 were synthesized and purified by HPLC (IDT, Coralville IA). Double-stranded DNA substrates were generated by mixing equimolar amounts of the complementary oligonucleotides, heating the mixture to 80°C, and allowing for slow cooling to room temperature for approximately 16 hours. Homopolymeric double-stranded oligos were analyzed by non-denaturing polyacrylamide gel electrophoresis (PAGE) and found to migrate exclusively as double-stranded 25 mers. All concentrations of DNA are stated in nucleotides (ssDNA) or base pairs (dsDNA). ATP was purchased as an HPLC purified solution (pH 7.5) (GE Healthcare). ATPγS (Sigma) was purchased in powdered form and resuspended in Tris base to generate a pH neutral solution.

### Oligonucleotide Strand Exchange Assays

5′-[^32^P]-labeled dsDNA was generated by incubating oligo 4 with T4 polynucleotide kinase (Invitrogen) and γ-[^32^P]-ATP for 1 hour at 37°C. The kinase was heat inactivated at 70°C for 10 min. Equimolar amounts of [^32^P]-labeled oligo 4 and unlabeled oligo 2 were added together in reaction buffer (20 mM Tris-HCl, pH 7.4, 50 mM NaCl, 3 mM MgCl_2_), heated to 80°C and allowed to anneal through slow cooling to room temperature. Strand exchange reactions were initiated upon addition of UvsX (2 µM) to 8 µM (nucleotides) single-stranded dA_25_ (oligo 4) and 2 µM double-stranded dA_25_:dT_25_ (oligo 4: oligo 2), and 2.5 mM ATP in reaction buffer. Reactions were conducted at room temperature. Aliquots were removed from the reaction and quenched at various time points in 50 mM EDTA, 1% SDS, 1X loading dye (Invitrogen). Samples were analysed using a 1X TBE 4–20% gradient native acrylamide gel (BioRad). The gel was dried under vacuum at 70°C for 45 min. Gels were visualized using the Bio-Rad Molecular Imager FX phosphorimaging system provided by the Vermont Cancer Center.

### UvsX Protein

UvsX protein was purified using a modification of previously published methods [22]. DNA encoding the UvsX protein was propagated in a pET27b expression vector (Novagen) and used to recombinantly overexpress UvsX protein in BL21 (DE3) *E.coli* cells (Stratagene). Cells were lysed via sonication and the soluble portion was obtained after centrifugation. This lysate was applied to a DEAE cellulose (Whatman) ion exchange column (column buffer 20 mM Tris-HCl, pH 7.4, 50 mM NaCl, 5 mM EDTA, 10% glycerol, 5 mM BME). Proteins were fractionated during elution from the column with a gradient application of column buffer containing 500 mM NaCl. Fractions containing UvsX were identified by SDS-PAGE analysis and applied to a hydroxylapatite column (Bio-Rad Laboratories) (column buffer 10 mM K_2_HPO_4_ pH 7.4, 100 mM NaCl, 10%glycerol, 5 mM BME). Proteins were fractionated during elution from the column with a gradient application of column buffer containing 700 mM K_2_HPO_4_ pH 7.4. The protein elutes from this column in both “early” and “late” eluting fractions. The “early” eluting fraction has no DNA binding or strand exchange activity. The late eluting fraction has these activities and was relatively homogeneous. This fraction was loaded onto a HiTrap Q HP (GE Healthcare) ion exchange column (column buffer 20 mM Tris-HCl, pH 7.4, 75 mM NaCl, 5 mM EDTA, 10% glycerol, 5 mM BME). Proteins were fractionated during elution from the column with a gradient application of column buffer containing 500 mM NaCl. Protein purity was analyzed by SDS-PAGE with Gel Code Blue (Thermo) protein stain. The protein was found to be greater then 95% pure, and was nuclease-free according to published criteria [23]. Protein was concentrated as necessary using Vivaspin centripetal concentrators (Sartorius Stedium), and stored at −80°C in storage buffer (20 mM Tris-HCl pH 7.5, 300 mM NaCl, 0.2 mM EDTA, 10% glycerol, 1 mM DTT). Protein concentrations were determined by absorption at 280 nm using an extinction coefficient of 69,760 M^−1^cm^−1^.

### Steady-state Fluorescence Measurements

Steady-state fluorescence measurements were made using a Quantamaster-6 fluorometer (PTI, Birmingham NJ). The excitation monochromator was set to 554 nm with 1 nm band pass slits. Spectra were taken from 560–600 nm with emission monochromator band pass slits set at 5 nm. Binding reactions contained 2 µM ssDNA (AlexaFluor 546-labeled oligo 1) or dsDNA (AlexaFluor 546-labeled oligo 1:oligo 2), 2 µM UvsX and 2.5 mM ATP in reaction buffer (20 mM Tris-HCl, pH 7.4, 50 mM NaCl, 3 mM MgCl_2_) at a total final volume of 80 µl at 25°C.

### Stopped-flow Fluorescence Measurements

Stopped-flow fluorescence measurements were made using an SX.18 MV stopped-flow fluorometer (Applied Photophysics, Leatherhead, Surrey, UK). Excitation monochromator was set at 550 nm with slits of 9.3 nm and a 570 nm long-pass filter was placed in front of the detector with slits of 9.3 nm. Protein, DNA, and nucleotide reagents in 20 mM Tris-HCl, pH 7.4, 50 mM NaCl, 3 mM MgCl_2_ reaction buffer, at 25°C, were mixed from 2 syringes according to the schematic provided with each figure. The fluorescently labeled species is indicated with an asterisk. Rapid mixing was in a 1∶1 ratio. At least 2 shots were averaged for each reaction and at least 3 independent reactions were averaged to generate the kinetic parameters. All binding reactions were monitored for at least 30 s. Just after mixing a decrease in fluorescence was observed indicative of binding, and after several seconds a lower resting level of fluorescence was observed indicating that equilibrium had been achieved (Figure S1). Reaction progress curves were fit to an exponential function (Graphpad) to determine the amplitude of fluorescence quenching at equilibrium, *I_b_* = (|*F_0_*-*F_b_*|), in which *F_0_* is the fluorescence intensity of the free DNA and *F_b_* is the fluorescence intensity of the DNA following addition of protein (both in arbitraty units). In reactions containing ATP and ssDNA, ATP hydrolysis catalyzed by UvsX eventually destabilizes UvsX-ssDNA interactions, resulting in loss of fluorescence quenching after a time delay. At earlier time points, however, these traces parallel those obtained in the presence of ATPγS (Figure S1). Therefore in these experiments the amplitude of fluorescence quenching was determined during the transient equilibrium period prior to the onset of signal recovery.

Amplitudes of fluorescence quenching were graphed as a function of UvsX concentration and fit to a quadratic binding equation (Equation 1) to determine an apparent equilibrium dissociation constant (*K_d_*) [24], [25] in which, *E_t_* is the total enzyme present, *B_t_* is the total DNA binding sites present, *I_f_* is the amplitude of fluorescence quenching of the free DNA (always floats close to zero), *I_b_* is the amplitude of fluorescence quenching of the bound DNA upon binding, *I_obs_* is the observed amplitude of fluorescence quenching at any given protein concentration. Equilibria were achieved within 30 seconds of mixing for all reactions for which apparent *K_d_* data are listed. This analysis assumes a 4 bp/UvsX binding site size. This binding site size has been measured for ssDNA in several other studies [16], [22] and for short dsDNA in this study (Figure S5).
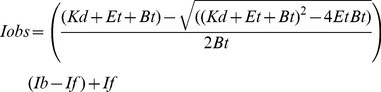
(1)


## Results

### Monitoring UvsX-DNA Interactions by the Quenching of AlexaFluor 546 Fluorescence

**Figure 1:**
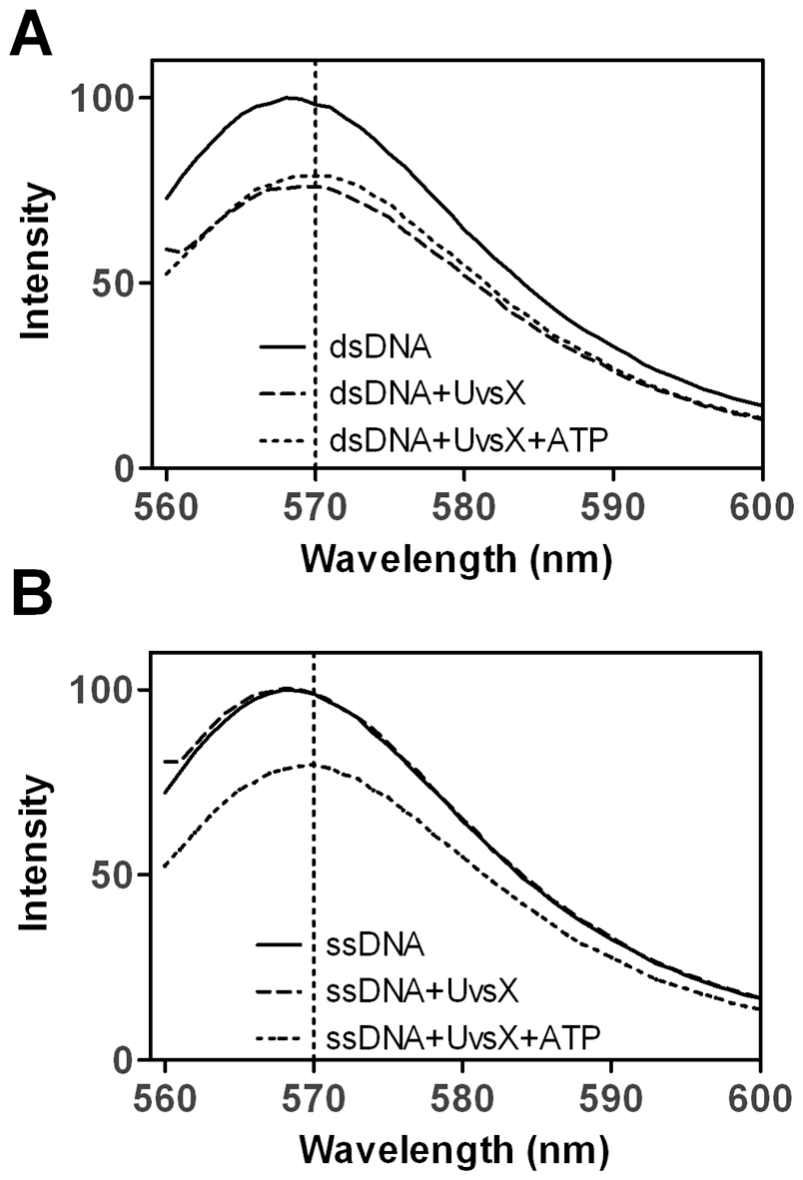
Effects of UvsX protein on fluorescence emission spectra of AlexaFluor 546-labeled oligonucleotides. Conditions were as described in Materials and Methods. The fluorescence emission spectra of (A) 2 µM (nucleotide pairs) double-stranded oligonucleotide dT_25_:dA_22_XA_2_ (oligo 2:oligo 1), and (B) 2 µM (nucleotides) single-stranded oligonucleotide dA_22_XA_2_ (oligo 1), were recorded in the absence of UvsX (solid line), in the presence of a saturating amount of UvsX (2 µM) (dashed line), and in the presence of saturating amounts of both UvsX and ATP (2.5 mM) (dotted line).

Previous studies established that the quenching of a DNA-bound fluorophore provides a reliable, quantitative read-out on the stability of UvsX-DNA interactions [20]. Here, oligonucleotide 1, dA_22_XA_2_, was labeled with AlexaFluor 546 as a probe to detect UvsX binding to DNA. A dsDNA substrate was generated by annealing the complimentary oligo 2 to the AlexaFluor 546 labeled oligo 1. Annealing of the complementary oligonucleotide did not change the fluorescence intensity of the labeled oligo 1 (free ssDNA and free dsDNA have the same fluorescence intensity). Non-denaturing PAGE analysis of the annealed product verified that it migrates exclusively as a duplex 25 mer (data not shown). Figure 1A shows the fluorescence emission spectrum of the free dsDNA substrate labeled with AlexaFluor 546 (2 µM nucleotide pairs) (solid line). The addition of UvsX (2 µM) generated a ∼25% quench in probe fluorescence with no spectral shift (dashed line). The addition of ATP (2.5 mM) did not affect the level of quenching appreciably, but the spectrum is slightly red-shifted (dotted line).

Similar methods were used to detect UvsX binding to ssDNA. Figure 1B shows the fluorescence emission spectra of labeled oligo 1 alone (solid line) (2 µM nucleotides). Addition of UvsX (2 µM) did not change the intensity of the emission (dashed line). However the addition of ATP (2.5 mM) caused a ∼25% decrease in the intensity of the fluorescence emission and a slight red-shift in the spectra indicative of UvsX binding (dotted line). Similar results were obtained when the position of the AlexaFluor 546 probe was moved to the 5′ end of the oligo (Figure S2). The data therefore indicate that stable binding to single-stranded oligo 1 is ATP-dependent under the conditions of our experiments. We have found that, like other recombinases, the binding of UvsX to ssDNA is influenced by the base composition of the polynucleotide [26]. Oligo 1 is essentially a dA homopolymer. UvsX binding to AlexaFluor 546-labeled oligo dT can be detected in the absence of ATP, however (Figure S3). This differential dependence on ATP is most likely due to differences in base stacking interactions in these sequences (see Discussion).

There was some concern that the presence of the AlexaFluor 546 label might alter the affinity of UvsX for labeled *versus* unlabeled DNA. Therefore we conducted experiments similar to those in Figure 1 using different ratios of labeled/unlabeled DNA. Results of these competition experiments indicate that the presence of the AlexaFluor 546 label on DNA does not significantly alter the DNA-binding affinity of UvsX (data not shown). Subsequent experiments make use of the fluorescence changes described in Figure 1 for quantitative analysis of UvsX-DNA interactions.

### UvsX Binding to dsDNA in the Presence of ATP and ATPγS

**Figure 2:**
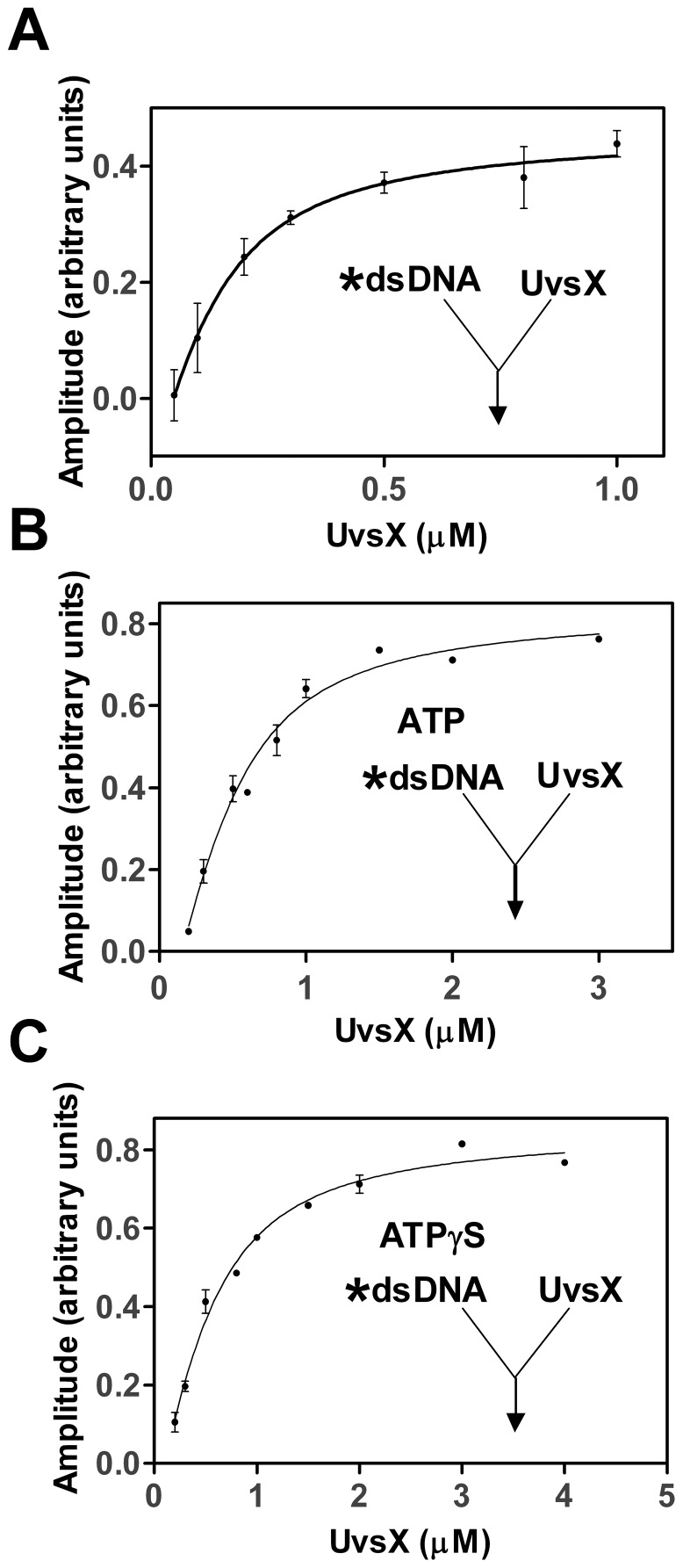
Binding of UvsX to double-stranded DNA in the absence or presence of nucleotide cofactors. Reactions were initiated by the addition of UvsX (final concentrations indicated on graph) to dsDNA, oligonucleotide dT_25_:dA_22_XA_2_ (oligo 2:oligo 1) and nucleotide cofactors final concentrations are indicated below. The amplitude of fluorescence quenching was graphed as a function of UvsX concentrations and fit to Equation 1 to determine an apparent *K_d_* value. (A) 0.5 µM (nucleotide pairs) dsDNA (B) 2 µM dsDNA and 2.5 mM ATP (C) 2 µM dsDNA and 900 µM ATPγS.

**Figure 3:**
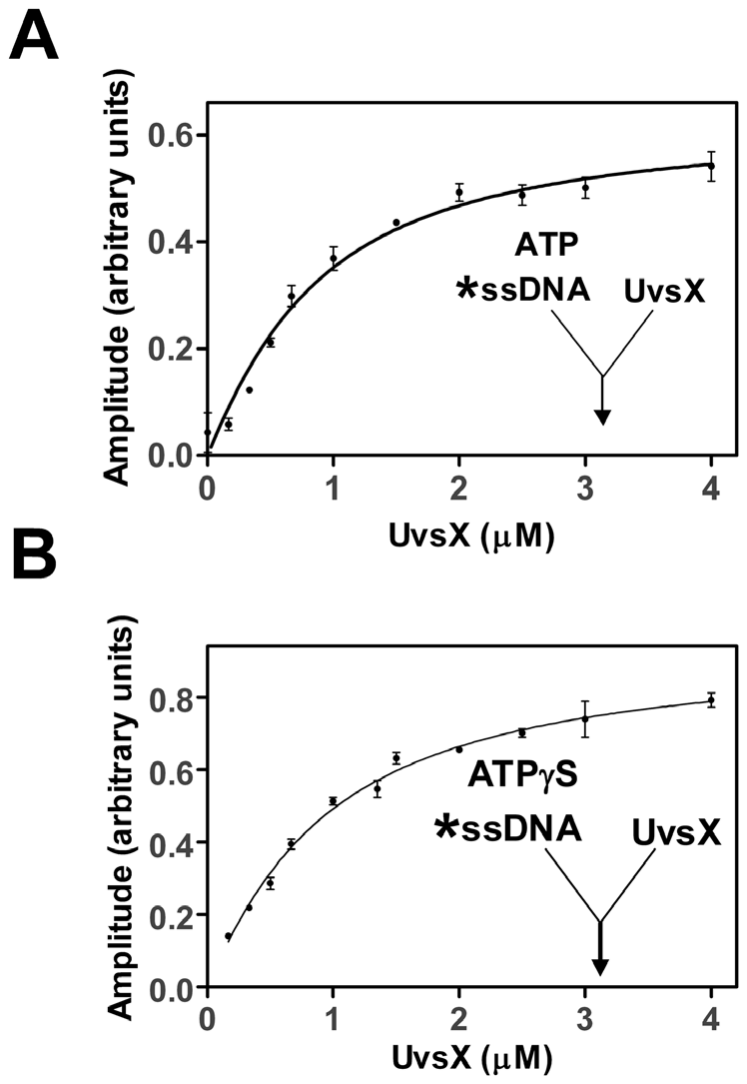
Binding of UvsX to single-standed DNA in the presence of ATP or ATPγS. Reactions were initiated by the addition of UvsX (final concentrations indicated) to a mixture of 2 µM (nucleotides) oligonucleotide dA_22_XA_2_ (oligo 1) ssDNA and (A) 2.5 mM ATP (final concentration) or (B) 900 µM ATPγS (final concentration). The amplitude of fluorescence quenching was graphed as a function of UvsX concentrations and fit to Equation 1 to determine an apparent *K_d_* value.

To allow for accurate comparisons with ssDNA binding data, fast mixing techniques were used to quantify UvsX-dsDNA interactions, even though dsDNA does not activate ATP hydrolysis by UvsX [22]. To measure binding affinity for the dsDNA substrate, various amounts of UvsX were incubated in one syringe and then rapidly mixed with 0.5 µM (nucleotides, final) AlexaFluor 546-labeled dsDNA. The amplitude of fluorescence quenching was graphed as a function of UvsX concentration (Figure 2A). These data were fit to Equation 1 to determine an apparent dissociation constant of 46±18 nM (Table 1). This analysis assumes a dsDNA binding site size of 4 bp, which was verified by stoichiometric titration under tight binding conditions (Figure S5), and which is equivalent to the ssDNA binding site size of 4 nucleotide residues [16], [17]. The use of the quadratic binding model in Equation 1 is justified by the observation that UvsX binding to short DNA molecules is not cooperative, as shown by the non-sigmoidal binding data in Figures 2–3. This feature of UvsX-DNA interactions is explored further in the Discussion.

**Table 1:** Apparent dissociation constants for UvsX binding to dsDNA or ssDNA^a^.

Other Ligands Present	Apparent *K_d_* (nM)	
	dsDNA	ssDNA
None	46±18	Not bound^b^
ATP	170±60	760±140
ATPγS	230±50	740±90
ATP+homologous ssDNA	1050±300	
ATP+heterologous ssDNA	N.D.^c^	
ATP+homologous dsDNA		≤750^d^

a Equilibrium binding data derived from Figures 2, 3, 4, and 6.

b No binding observed under the experimental conditions used.

c Not Determined. Measuring the apparent *K_d_* for dsDNA in the presence of ATP and heterologous ssDNA requires unattainably high protein concentrations.

d Based on observation in Figure 4 that homologous dsDNA does not destabilize UvsX-ssDNA interactions.

In Figure 1A, ATP was not required for UvsX binding to dsDNA, however a slight red shift was observed in the presence of ATP. This may indicate that ATP is bound under these conditions and that it may affect the binding affinity or conformation of the UvsX filament on dsDNA. Similar assays as those described above were performed to measure the binding affinity for UvsX to dsDNA in the presence of ATP or ATPγS. Absolutely higher concentrations of both UvsX and dsDNA were required to measure accurately this elevated apparent *K_d_*. The concentrations of ATP and ATPγS used in these experiments were saturating as determined in a separate assay (Figure S4). To determine the binding affinities various amounts of UvsX were rapidly mixed with 2 µM (final, base pairs) of the labeled duplex and either ATP (2.5 mM) or ATPγS (900 µM). The amplitude of fluorescence quenching was graphed as a function of protein concentration (Figure 2B–C). These data were fit to Equation 1 to determine apparent *K_d_* values of 170±60 nM (ATP) and 230±50 nM (ATPγS), respectively. Based on these values, it is evident that UvsX binds to dsDNA with 4 to 5-fold reduced affinity in the presence of ATP or ATPγS (Table 1).

### UvsX Binding to ssDNA in the Presence of ATP and ATPγS

Similar rapid mixing assays were performed to determine the binding affinities of UvsX to ssDNA in the presence of substrate ATP or substrate analog ATPγS. A previous study established that UvsX hydrolyzes ATPγS very slowly in the presence of ssDNA [27]. The amounts of nucleotide cofactor used were saturating as determine in a separate assay (Figure S4). Various amounts of UvsX were incubated in one syringe and then rapidly mixed with 2 µM (nucleotides, final) labeled oligo 1 and 900 µM ATPγS (final) or 2.5 mM ATP (final). The amplitude of fluorescence quenching was graphed as a function of UvsX concentration (Figure 3A–B). These data were fit to Equation 1 to determine apparent *K_d_* values of 740±90 nM in the presence of ATPγS and 760±140 nM in the presence of ATP (Table 1). The similarity of these two dissociation constants in the presence of these two nucleotide cofactors indicates that our methods have most likely captured the dissociation constant of the ATP bound species before hydrolysis. Consistent with this, results of coupled ATPase assays indicate that ADP release is not detectable on the time scale of the reactions in Figure 3 (≤30 s) (data not shown). These observations are important because the ATP bound species is transient but it is also thought to be the form of the enzyme that is critical for homology search [28], [29]. This will be a key factor in later experiments when homologous and heterologous substrate pairs are present simultaneously.

### UvsX Binding to ssDNA in the Presence of ATP is Unaffected by Homologous dsDNA

The apparent *K_d_* values measured for ssDNA in the presence of ATP/ATPγS are 3–4 fold greater than those measured for dsDNA under identical conditions (Table 1). This may be counterintuitive since it is thought that the recombinase filament must form on ssDNA to initiate strand exchange properly. However these apparent *K_d_* values were only determined in the presence of one polynucleotide substrate or the other. To more closely mimic DNA strand exchange conditions, we measured the affinity of UvsX for ssDNA and dsDNA substrates when they are present simultaneously. The reaction can be monitored from the perspective of each oligonucleotide, by placing the AlexaFluor 546 probe on either the dsDNA or the ssDNA. Then how the presence of one substrate, at one site on the enzyme, may affect binding of the other substrate at another site on the enzyme can be determined. All reactions were conducted in the presence of a saturating amount of ATP.

AlexaFluor 546-labeled ssDNA and unlabeled dsDNA were used to measure ssDNA binding in the presence of increasing dsDNA concentrations. The binding of UvsX to labeled ssDNA was monitored under conditions in which the ssDNA is saturated with UvsX (see Figure 3A) and unlabeled dsDNA of homologous sequence is titrated in. UvsX was simultaneously mixed with both DNA substrates and a saturating amount of ATP. Figure 4 shows 3 µM UvsX (final) binding to 2 µM labeled ssDNA (final, nucleotides) in the presence of 2.5 mM ATP and increasing amounts of homologous dsDNA. Reaction progress curves were fit to an exponential function to determine the amplitude of fluorescence quenching. The amplitude was graphed as a function of homologous dsDNA concentration and data were fit to a line to demonstrate the trend (solid line). There is no change in the amplitude of fluorescence quenching in the presence of ever increasing amounts of homologous dsDNA, indicating that ssDNA binding is not destabilized in the presence of dsDNA. These results suggest that the ssDNA and dsDNA substrates do not directly compete for the same binding site on UvsX. However the possibility still exists that ssDNA binding is not destabilized in the presence of homologous dsDNA because the dsDNA, which is not labeled in this experiment, is not bound. This possibility is eliminated in subsequent experiments.

**Figure 4:**
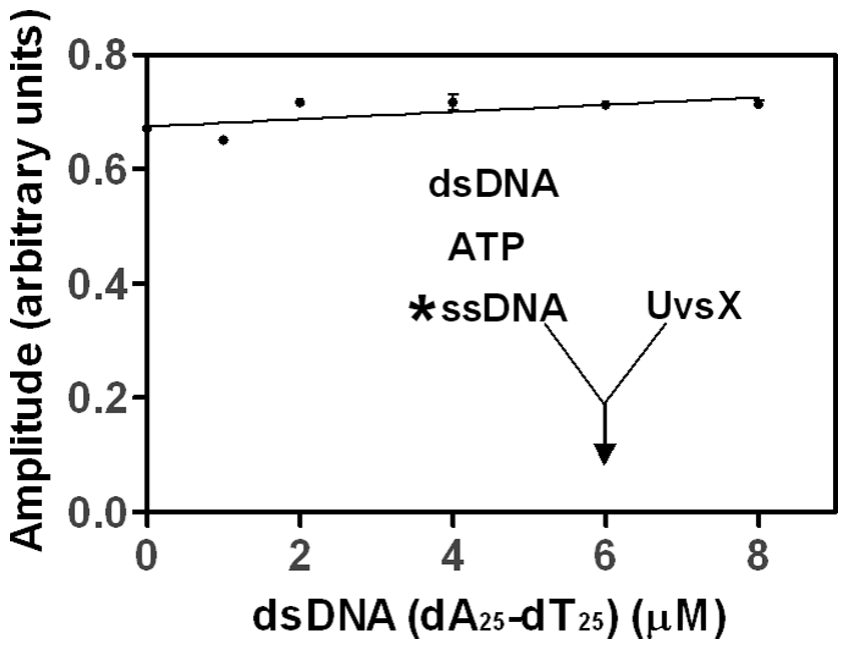
Binding of UvsX to ssDNA in the presence of increasing amounts of homologous dsDNA 3 µM UvsX (final concentration) was added to mixtures containing final concentrations of 2 µM ssDNA (dA_22_XA_2_, oligo 1), 2.5 mM ATP, and various concentrations of homologous dsDNA (dT_25_:dA_25_, oligo 2:oligo 4). The amplitude of fluorescence quenching was graphed as a function dsDNA and a line was fit (solid line) to demonstrate the trend.

### UvsX Binding to dsDNA is Destabilized in the Presence of ATP and ssDNA of Homologous Sequence

**Figure 5:**
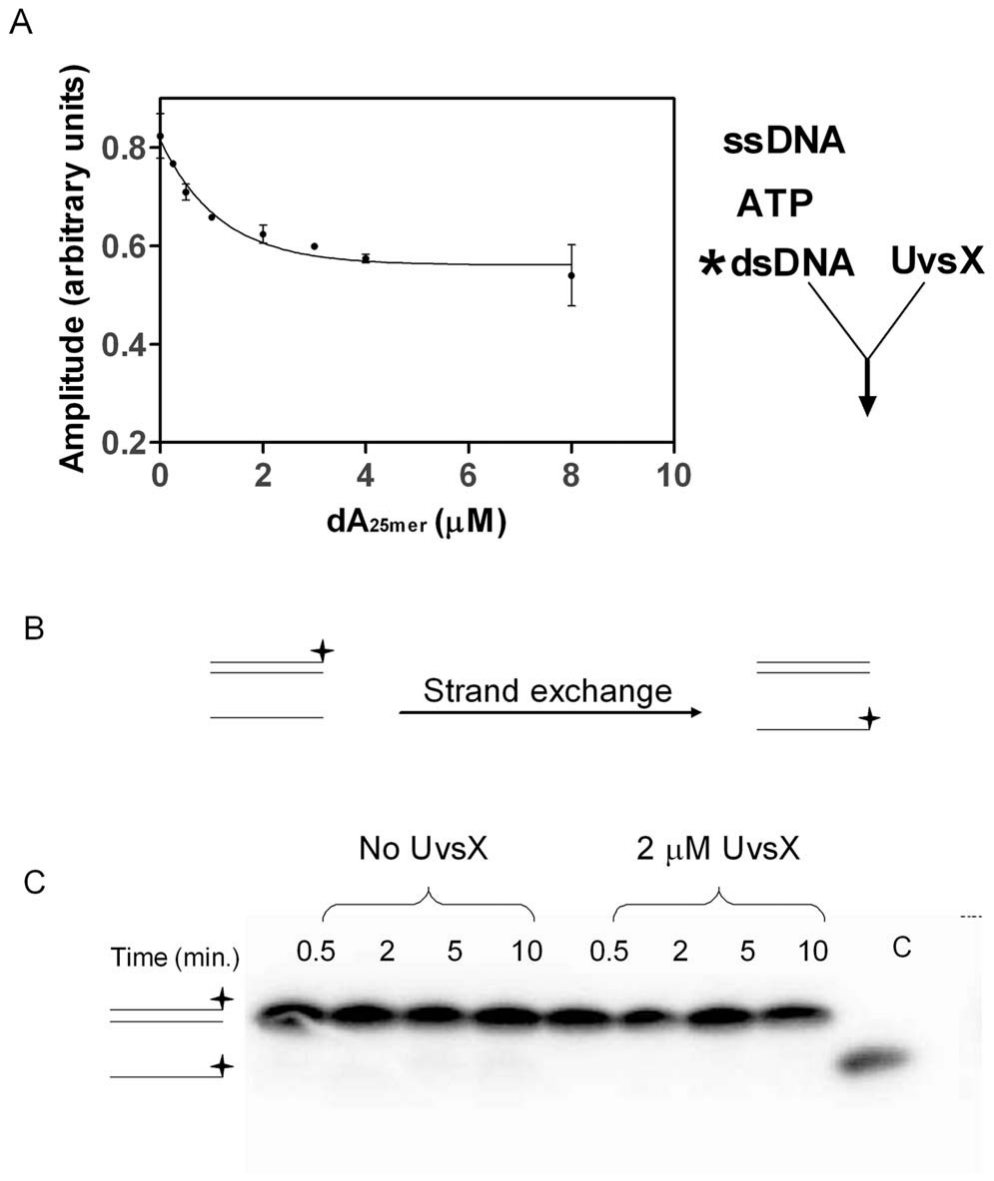
Binding of UvsX to dsDNA in the presence of increasing amounts of homologous ssDNA. A) 2 µM UvsX (final concentration) was added to a mixture of 2 µM dsDNA (dT_25_:dA_22_XA_2_ oligo 2:oligo 1), 2.5 mM ATP, and various amounts of homologous ssDNA (dA_25,_ oligo 4). The amplitude of fluorescence quenching was graphed as a function of ssDNA concentration and fit to a single exponential function (solid line) to demonstrate the trend. B) Schematic of strand exchange reaction used to determine if strand exchange is occurring during the monitoring of binding reactions depicted in A. C) Strand exchange reaction containing 0 or 2 µM UvsX, 8 µM ssDNA (dA_25_) and 2 µM ^32^-P labeled dsDNA (dA_25_:dT_25_) in the presence on 2.5 mM ATP. Control lane “C” indicated where on the gel the outgoing strand would be seen if strand exchange were to occur.

**Figure 6:**
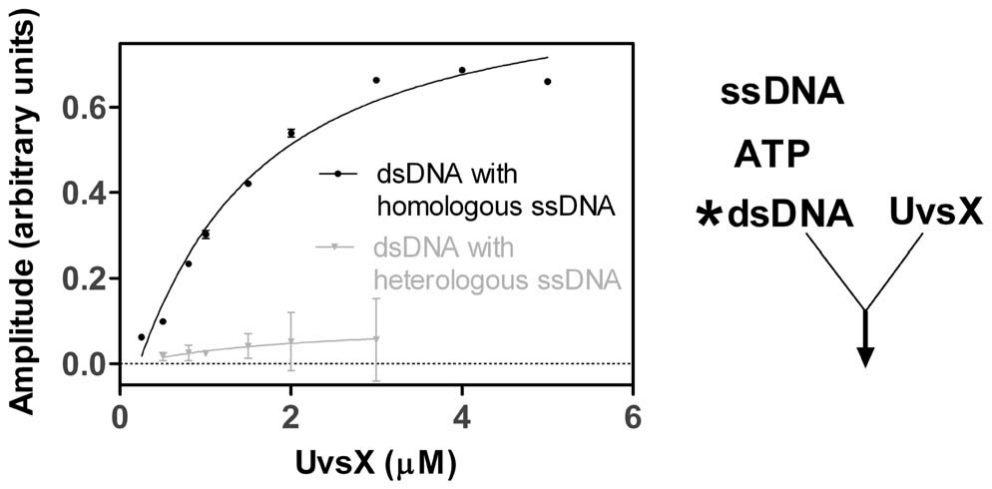
Binding of UvsX to dsDNA in the presence of excess homologous or heterologous ssDNA. Various amounts of UvsX were added to mixtures containing final concentrations of 2 µM dsDNA (dT_25_:dA_22_XA_2_ oligo 2: oligo 1), 2.5 mM ATP, plus 40 µM of either homologous (dA_25,_ oligo 4) or heterologous (dC_25_ oligo 3) ssDNA. The amplitude of fluorescence quenching was graphed as a function of UvsX concentration. Data for homologous ssDNA were fit to Equation 1 to determine an apparent *K_d_* value (black line). Data for heterologous ssDNA were fit to a line (gray line).

**Figure 7:**
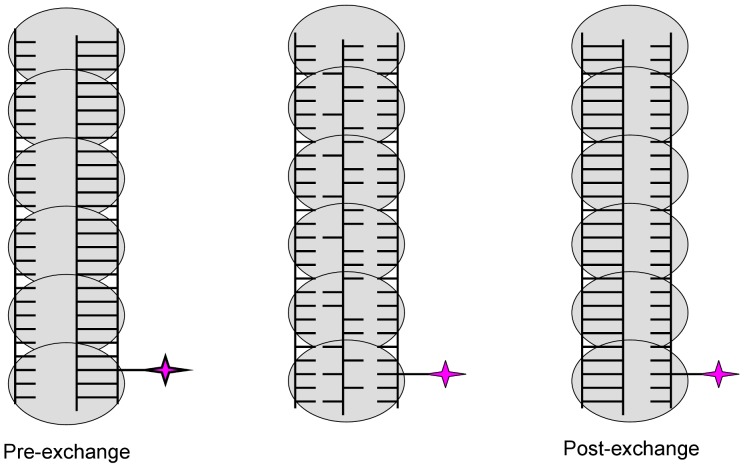
Potential structures of the three-stranded intermediate formed in reactions in which homologous substrate sets were used in the presence of ATP. This figure shows the three intermediates that could form upon mixing a fluorescent dsDNA with homologous ssDNA and ATP. The three-stranded intermediate formed may be pre-strand exchange, post-strand exchange, or an intermediate. See Discussion for further information.

Similar reactions as those above were conducted to determine if dsDNA binding is changed in the presence of ssDNA. Labeled dsDNA and unlabeled ssDNA were used to measure dsDNA binding in the presence of increasing ssDNA concentrations. The binding of UvsX to AlexaFluor 546-labeled dsDNA was monitored under conditions in which the dsDNA is saturated with UvsX (see Figure 2B) and unlabeled ssDNA of homologous sequence is titrated from 0 µM to 8 µM. UvsX was mixed with both DNA substrates and a saturating amount of ATP simultaneously. Reaction progress curves were fit to an exponential function to determine the amplitude of fluorescence quenching. The amplitude was graphed as a function of ssDNA concentration (Figure 5A). When no ssDNA was present the amplitude reflects saturated binding of UvsX to dsDNA substrate (compare to Figure 2B). Increasing amounts of ssDNA generated decreased amplitudes, suggesting less binding of dsDNA (Figure 5A). It could be argued that the observed changes in Figure 5A are caused by a simple competition between ssDNA and dsDNA for binding to a single site in the UvsX filament. However this possibility is ruled out by the data in Figure 4, which show that dsDNA does not displace bound homologous ssDNA from the filament. Another possibility is that the decreased amplitudes are generated as a result of strand exchange occurring causing the labeled strand to be displaced. We have also ruled out this possibility by monitoring DNA strand exchange under identical conditions using a [^32^P]-labeled oligonucleotide in the place of the AlexaFluor 546–labeled strand of the duplex (Figure 5B). Reactions were carried out at the highest concentration of ssDNA used in Figure 5A. As seen on the gel in Figure 5C, no strand exchange occurs during the time in which these assays were conducted. Therefore the decrease in fluorescence observed in Figure 5A is indicative of a decrease in dsDNA binding and reflects an increased apparent *K_d_* value for dsDNA in the presence of homologous ssDNA. The labeled species added to this reaction was dsDNA however at equilibrium the labeled species may be part of a strand exchange intermediate and no longer strictly “double-stranded” (see Figures 6, 7). The resulting apparent *K_d_* measurements discussed below, although strictly discussed as the apparent *K_d_* for dsDNA in the presence of homologous ssDNA, may actually be reflective of a 3 strand intermediate.

To determine the value of this increased apparent *K_d_*, a titration of UvsX onto labeled dsDNA in the presence of excess unlabeled homologous ssDNA was performed (Figure 6). This experiment is identical to the experiment shown in Figure 2B with the exception that excess homologous ssDNA is included. Therefore in contrast to experiments shown in Figure 2B, this experiment measures the apparent *K_d_* for dsDNA binding when both the ATP and the ssDNA binding sites are occupied. Various amounts of UvsX were added to 2 µM labeled dsDNA (AlexaFluor 546-labeled oligo 1:oligo 2) in the presence of 2.5 mM ATP and 40 µM unlabeled ssDNA of homologous sequence (oligo 4). UvsX was mixed simultaneously with both DNA substrates and ATP. The reaction progress curves were fit to an exponential function to determine the amplitude of fluorescence quenching, which was then graphed as a function of UvsX concentration (Figure 6, black circles). These data were fit to Equation 1 to determine an apparent *K_d_* of 1.05±0.3 µM (solid line). Figure 6 (black circles) shows a binding curve similar to that of dsDNA binding alone (Figure 2B) in that the final amplitude of fluorescence quenching is the same. However more UvsX is required to reach a fully quenched state indicating that changes in dsDNA binding in the presence of homologous ssDNA are predominately due to an increase in the apparent *K_d_* value for homologous dsDNA binding to a UvsX-ssDNA complex when compared to binding of dsDNA to UvsX alone (Table 1). Alternatively, since these reactions in principle contain the minimum requirements for a strand exchange reaction to occur (UvsX, Mg^2+^ATP, ssDNA, and homologous dsDNA), the observed changes in apparent *K_d_* could reflect a change in affinity for substrates vs. products. The data in Figure 5C appear to rule this out, however, since they establish that complete strand exchange does not occur over a time scale of 0–10 minutes, whereas all of the binding measurements to determine apparent *K_d_* values in Table 1 are taken during the first 30 seconds after mixing. Therefore the data in Figure 6 (black circles) are consistent with the formation of an intermediate complex containing three DNA strands bound simultaneously to UvsX (see Discussion and Figure 7).

### UvsX Binding to dsDNA in the Presence of ATP and ssDNA of Heterologous Sequence

Analogous experiments were conducted to investigate the effects of heterologous ssDNA on the stability of UvsX-dsDNA interactions (Figure 6 gray circles). In these experiments, heterologous ssDNA (oligo 3) was substituted for homologous ssDNA (oligo 4), while the labeled dsDNA substrate (AlexaFluor 546-labeled oligo 1:oligo 2) was the same in both data sets depicted in Figure 6. All other conditions were identical. Differences observed in dsDNA binding were due to changes in the unlabeled ssDNA present. The equilibrium data demonstrate a dramatic decrease in binding to dsDNA in the presence of heterologous ssDNA (Figure 6, gray circles). The apparent *K_d_* value for dsDNA under these conditions was too high to be measured at experimentally attainable protein concentrations.

## Discussion

Results of this study suggest that UvsX recombinase avoids inhibition by excess non-homologous dsDNA through allosteric effects mediated by ssDNA binding. In the presence of ssDNA, dsDNA binding is attenuated and correlated with its homology to the bound ssDNA. These insights were made possible by a novel assay that detects the binding of dsDNA to a site on UvsX when ssDNA occupies a second site, or vice-versa.

### AlexFluor 546 as a Probe for UvsX-DNA Interactions

The AlexaFluor 546-DNA conjugate was used as a fluorescence probe for UvsX-DNA interactions. This probe offers several advantages including: sufficient brightness for work at nanomolar concentrations; similar fluorescence signals in ssDNA and dsDNA; no effect on UvsX-DNA binding affinities. A previous study employed fluorescein-labeled oligonucleotides as quantitative probes for UvsX-ssDNA interactions [20]. The AlexaFluor 546-DNA conjugates provide a similar utility, with the added advantage of probe brightness, a larger amplitude of fluorescence quenching by UvsX, and therefore greater sensitivity. Other studies employed etheno-modified ssDNA containing the fluorescent bases ethenoadenine and ethenocytosine [16], [30], [31]. These probes provide detailed information on the structural changes (unstacking, extension) of ssDNA as it is bound by a recombinase. However ethenobases are known to alter the affinity of recombinase-ssDNA interactions, and they disrupt base pairing, preventing their use in dsDNA or in homologous pairing reactions [16], [32]. The AlexaFluor 546 probe avoids this problem and thus allows direct comparisons of ssDNA and dsDNA binding by UvsX, and of the effects of one bound lattice on the binding of the other.

Several observations suggest that general environmental features within recombinase-DNA filaments are responsible for the quenching of AlexaFluor 546 fluorescence, and that signal is relatively insensitive to filament conformation. Probe fluorescence is insensitive to the ss/ds character of the DNA it is attached to, and UvsX binding quenches probe fluorescence to a similar degree on either substrate (Figure 1). Similar amplitudes of fluorescence quenching are observed when the probe attachment position on the oligo is changed or when the oligo sequence is changed (Figures S2, S3). Therefore the quenching of probe fluorescence appears to be a simple indicator of the occupancy of DNA by the recombinase. This is supported by the observation that UvsX quenches the fluorescence of AlexaFluor 546-labeled oligo dT in the absence or presence of nucleotide (Figure S3), which rules out the possibility that nucleotide-free UvsX could somehow occupy ssDNA without quenching the probe.

### A minimalist System for the Sensing of Sequence Homology by UvsX Recombinase

The results of this study provide a framework for understanding the homologous pairing activity of a recombinase through the coordination of its ssDNA and dsDNA binding activities. To lower the complexity of the problem, a minimalist system was devised for the sensing of sequence homology or heterology by UvsX recombinase. Homopolymeric DNA substrates were used to avoid regions of secondary structure in ssDNA, which could bind the recombinase differentially, and to generate uniformly homologous or heterologous substrate sets. Short oligonucleotides were used to simplify DNA binding analyses by minimizing cooperativity. Binding isotherms in Figures 2–3 are non-sigmoidal, and binding kinetics have no lag phase (Figure S1 and data not shown), indicating that UvsX binds non-cooperatively to short oligos. This is likely due to the fact that UvsX exists in oligomeric structures on the order of hexamers, which may bind as a unit to short oligos (J. Liu and S. Morrical, unpublished results). The 25-mers used in our experiments were designed to accommodate six subunits of UvsX, equivalent to one hexamer or one helical turn of the presynaptic filament. Cooperative binding observed on long ssDNA molecules [16], [17] likely involves longer-range interactions between oligomeric units of UvsX.

The apparent binding constants reported in Table 1 may be biased by the homopolymeric sequences and short lengths of these substrates compared to more physiological DNA substrates. However the significance of this work lies in the demonstration of coordination between the ligand binding sites of UvsX (ssDNA, dsDNA, ATP) rather than in the absolute binding constants for the model DNA lattices. This coordination is evident from the changes seen in apparent *K_d_* values for the same substrate at the same site as the occupancy of the other sites is changed (Table 1). The information obtained here using minimalist DNA substrates is relevant to UvsX interactions with more physiological DNA substrates. The affinity preference of UvsX for homopolymeric duplex over ssDNA is consistent with previous findings that: (1) UvsX binds preferentially to mixed sequence dsDNA over ssDNA [21]; and (2) The intrinsic affinity parameter (independent of cooperativity) of UvsX for dsDNA equals or exceeds that for ssDNA over a wide range of salt concentrations (H. Xu and S. Morrical, unpublished results). These findings indicate that under physiological conditions (high dsDNA/ssDNA ratio, relatively high ionic strength) UvsX filaments would preferentially form on dsDNA in the absence of other factors.

The minimalist, oligonucleotide-based recombination system allows for homology detection by UvsX (Figure 6), however complete strand exchange is not observed (Figure 5C). As depicted in Figure 7, the three-stranded species detected likely mimics a homologous pairing intermediate that forms prior to the ejection of the outgoing strand from the complex. It is significant that the formation of this species depends on the sequence homology of the strands present, however our data cannot distinguish between two possible forms of this complex: the pre- and post-strand exchange forms (Figure 7). The quenched intermediate may in fact be a post-strand exchange complex (Figure 7). Complete ejection of the outgoing strand may not occur under our experimental conditions due to the absence of accessory proteins such as Gp32, which stimulates UvsX-catalyzed strand exchanges between long homologous DNA substrates in part by sequestering the outgoing strand [33], or UvsW helicase, which stimulates strand transfer by promoting branch migration [34]. Alternatively, failure to exchange strands could be due to insufficient filament length or to effects of the homopolymeric DNA sequences used in our experiments.

### Differential Effects of ATP on UvsX-ssDNA and –dsDNA Interactions

In the presence of nucleoside triphosphates, recombinases including UvsX bind and stretch ssDNA to form an active filament conformation. Recombinase binding and formation of the stretched conformation is somewhat sequence dependent due to differential base stacking interactions of the four different bases [26]. Formation of the stretched conformation is more favorable within tracts of sequence in which base stacking interactions are low (e.g. polypyrimidine tracts). In this study we have employed the fluorescently labeled ssDNA homopolymer dA_22_XA_2_ (oligo 1). This homopolymer is structured and has high base stacking energy when compared with other sequences, such as poly(dT) [35], [36] and thus the recombinase may bind to it with less affinity due to the fact that attaining the stretched conformation requires overcoming more base stacking energy. This could explain our observation that UvsX binding to dA_22_XA_2_ requires ATP or ATPγS under the conditions of our experiments (Figures 1–3). As noted previously, binding to oligo dT, a lattice with lower base stacking energy, is not ATP-dependent (Figure S3). In this respect oligo 1 is a stringent mimic of ssDNA that is encountered in the cell.

While ssDNA binding is enhanced by ATP or ATPγS, we observe that dsDNA binding is destabilized by these nucleotides (Table 1). Much like ssDNA, dsDNA within the ATP containing filament may be stretched. Previously, the low-resolution structures of UvsX-dsDNA filaments were solved in the presence of ADP-AlF_4_ or of ADP by cryo-electron microscopy [18]. The ADP-AlF_4_ structure was stretched by 150% compared to the structure in the presence of ADP. Recently an X-ray crystal structure of a truncated form of UvsX was solved [34]. This atomic structure was modeled into the UvsX filament structures solved by cryo-electron microscopy. The investigators report that a nucleotide cofactor cannot be accommodated in the condensed filament [34]. These findings suggest that the low-*K_d_* form of UvsX-dsDNA that we observe in the absence of ATP represents a condensed filament, while the high-*K_d_* form seen in the presence of ATP represents a stretched filament.

### Mobilization of UvsX from dsDNA in Response to ssDNA

**Figure 8:**
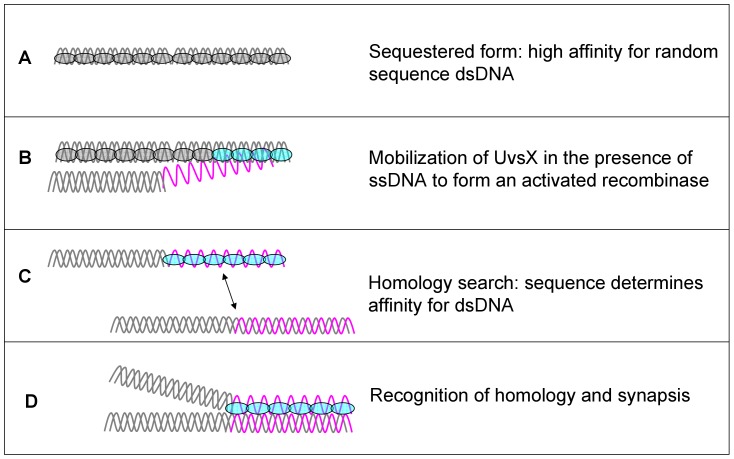
Model for ssDNA recognition and activation of recombination in the presence of excess dsDNA. (A) In the presence of only dsDNA, UvsX has high affinity for dsDNA and low turnover of ATP. (B) In the presence of both ssDNA and dsDNA UvsX has higher affinity for ssDNA (C) and affinity for dsDNA is directly correlated to its homology to the ssDNA bound by the enzyme. (D) The ssDNA bound by the enzyme dictates the search for homology within the dsDNA. See text for details.

The binding of UvsX to ssDNA is necessary to activate its catalytic activities. The vast majority of DNA in the T4-infected cell is double-stranded, and has the potential to be a potent inhibitor of UvsX facilitated recombination. In fact UvsX binds with greater affinity to dsDNA alone than to ssDNA alone (Table 1; [21] and unpublished results). Binding to dsDNA does not activate the ATPase activity of UvsX [22] (data not shown). This lack of ATP hydrolysis is not due to a lack of ATP binding, since binding to dsDNA is weakened in the presence of ATP (Table 1). The apparent *K_d_* of the UvsX-dsDNA complex is still relatively low (ca. 200 nM) in the presence of ATP however, suggesting that the enzyme seldom exists in the DNA-free form. We propose instead that UvsX-dsDNA complexes represent a reservoir or sequestered form of inactive recombinase, which avoids the nonproductive consumption of ATP until recombinagenic ssDNA is generated (Figure 8).

The data in Table 1 indicate that ATP-induced affinity changes are insufficient to favor a simple exchange of UvsX from dsDNA to ssDNA. Instead, pre-formed UvsX-dsDNA complexes appear to be actively remodeled by ssDNA. The presence of heterologous ssDNA dramatically destabilizes UvsX-dsDNA binding, while the presence of homologous ssDNA raises the apparent *K_d_* for dsDNA by approximately 5-fold (Figure 6, Table 1). It is clear from the data that the homologous ssDNA and dsDNA molecules do not compete for binding to a single site within the UvsX filament (Figures 4 and 5). Instead the data favor a model in which ssDNA binding allosterically alters the apparent *K_d_* for dsDNA binding to a second site within the UvsX filament. Together, the binding data are consistent with a model in which UvsX is tightly associated with dsDNA when recombination is not occurring in the cell (Figure 8). When present on dsDNA, UvsX can bind but not hydrolyze ATP. ssDNA generated as a result of DNA damage binds to the duplex-bound UvsX, activating it for recombination and diminishing its affinity for heterologous dsDNA. Thus, if duplex-bound UvsX can be thought of as a reservoir or sequestered form of inactive recombinase, the generation of ssDNA mobilizes the enzyme, releasing UvsX from unproductive association with random dsDNA while “tuning” the enzyme to discriminate between dsDNA sequences on the basis of homology to the ssDNA present (Figure 8). The transfer of UvsX from dsDNA to ssDNA is necessary to activate ATP hydrolysis and exchange, which in turn facilitates conformations of the filament that promote D-loop formation upon recognition of homologous DNA.

### Accessory Proteins may also Mitigate Potential Inhibitory Effects of dsDNA

While UvsX itself appears to have the ability to clear itself from potentially inhibitory dsDNA in response to a ssDNA signal, other proteins are also needed to form and/or stabilize an active presynaptic filament, and these proteins may also help to confer to UvsX a selective affinity for ssDNA. UvsY, a recombination mediator protein required for UvsX recombination transactions in vivo, has a strong bias toward binding to ssDNA and may promote the selective nucleation and stabilization of UvsX filaments on ssDNA [9], [20], [37]. UvsX and UvsY work in concert with Gp32, which plays an important role in presynaptic filament assembly by denaturing inhibitory secondary structure in ssDNA before being displaced by UvsX/UvsY [11], [38]. These two accessory proteins together with the intrinsic coordination of the two DNA binding sites of UvsX demonstrated here, may help stabilize active UvsX filaments on ssDNA once it has been mobilized from association with random dsDNA in an unproductive/sequestered complex.

*In vitro* DNA strand exchange assays with many recombinases are typically staged in such a way that the recombinase filament is pre-assembled on ssDNA before dsDNA is introduced into the reaction. Physiologically, recombinases almost always encounter substrates in the opposite order–dsDNA before ssDNA. Eukaryotic organisms appear to rely on the DNA translocase activity of Rad54 to remove Rad51 recombinase from random dsDNA as a prerequisite to presynaptic filament assembly on ssDNA [39]. Rad51-dsDNA complexes turn over very slowly in the absence of Rad54, leading to inhibition of strand exchange due to excess duplex [40]. In the T4 recombination system, UvsX-dsDNA complexes appear to turn over efficiently in response to ssDNA, circumventing the need for a Rad54-like DNA translocase activity. T4 relies on homologous recombination events to initiate genomic replication during late stages of infection in E. coli cells, resulting in a greatly amplified phage burst [11], [41]. The enhanced dynamics of UvsX-DNA interactions allows the phage to rapidly mobilize its recombination machinery to facilitate recombination-dependent replication.

## Supporting Information

Figure S1 **Typical traces of fluorescence quenching data used to measure dissociation constants for UvsX and labeled DNAs in the presence of nucleotide cofactors.** UvsX hydrolyzes ATP in the presence of ssDNA. ATP hydrolysis is associated with the release of ssDNA. Thus the UvsX:ATP:ssDNA tripartite species is transient. In order to measure the affinity of UvsX for ssDNA in the presence of ATP rapid mixing techniques were used to observe ssDNA binding before ATP hydrolysis. We also used ATPγS, an ATP analogue which is hydrolyzed slowly. Reactions were initiated by the addition of UvsX (final concentrations indicated) to a mixture of 2 µM (nucleotides) ssDNA and (A) 2.5 mM ATP (final concentration) or (B) 900 µM ATPγS (final concentration). Reaction progress was monitored for up to 1000 s however only the first 30 s were used to determine the binding constants (Table 1). These data were fit to an exponential function to determine the total amplitude of quenching at each protein concentration. These amplitudes were then plotted as a function of UvsX concentration and these data were fit to Equation 1 to determine an apparent *K_d_*. The similarity of the ATP and ATPγS fluorescence data as well as the similarity of the apparent *K_d_* values obtained lead us to conclude that our techniques allowed us to measure the ATP bound form of the enzyme in the presence of ssDNA.
(TIF)

Figure S2 **Effects of UvsX protein on fluorescence emission spectra of AlexaFluor 546-labeled oligonucleotide with alexafluor 546 differentially positioned.** A single-stranded 25 mer oligonucleotide of the sequence dA_25_ with a 5′ C6 amino-modifier was covalenty labeled with AlexaFluor 546. The fluorescence emission from 560–600 nm with an excitation of 554 nm was recorded for 2 µM of this oligonucleotide alone (solid line), after the addition of 1.35 µM UvsX (dashed line) and after the addition of 3 mM ATP (dotted line). Reaction conditions were as described in the material and methods section for steady-state measurements.
(TIF)

Figure S3 **UvsX binding to 25** **mer oliognucleotide 5′-dT_22_XT_2_-3′, in which X is amino-modifier C2 dT where the alexa 546 probe is covalently attached.** 2uM ssDNA was added to various amounts of UvsX in the presence and absence of 900 µM ATPγS. The (A) fluorescence quenching relative to ssDNA alone and the (B) amplitude of fluorescence quenching was graphed as a function of UvsX concentration.
(TIF)

Figure S4 **Titration of nucleotide cofactors to determine saturated binding conditions.** A nucleotide cofactor is required for UvsX to binding to single-stranded oligo 1. ATP and ATPγS titrations were conducted to determine a saturating amount of ATP and ATPγS to be used in the ssDNA binding assays. Rapid mixing techniques with a SX.18 MV stopped-flow fluorometer (Applied Photophysics, Leatherhead, Surrey, UK) were used to measure binding of UvsX to ssDNA in the presence of the nucleotide cofactor before hydrolysis. 2 µM AlexaFluor 546 labeled oligo 1 and 1.35 µM UvsX were rapidly mixed with 0–3 mM ATP or ATPγS in a reaction buffer containing 20 mM Tris-HCl, pH 7.4, 50 mM NaCl, 3 mM MgCl_2_. The amplitude of fluorescence quenching was graphed as a function of ATP or ATPγS concentration. From these data 900 µM ATPγS and 2.5 mM ATP were chosen as saturating concentrations used to measure DNA binding by UvsX.
(TIF)

Figure S5 **Stoichimetric binding of UvsX to dsDNA.** Various amounts of UVSX was added to 2 µM (nucleotide pairs) dsDNA in the absence of a nucleotide cofactor. Three separate experiments were conducted as specified in the material and methods section. Total change in fluorescence was graphed as a function of UvsX concentration. Data were fit to Equation 1 (solid line). The binding site size indicated by these data is 4 nucleotide pairs/UvsX monomer (2 µM nucleotide pairs is saturated by 0.5 µM UvsX).
(TIF)
