# Supplementary figures and images for: Coordinated Binding of Single-Stranded and Double-Stranded DNA by UvsX Recombinase
Source: PLoS One. 2013 Jun 18;8(6):e66654. doi: 10.1371/journal.pone.0066654 (PMC3688935; doi:10.1371/journal.pone.0066654)

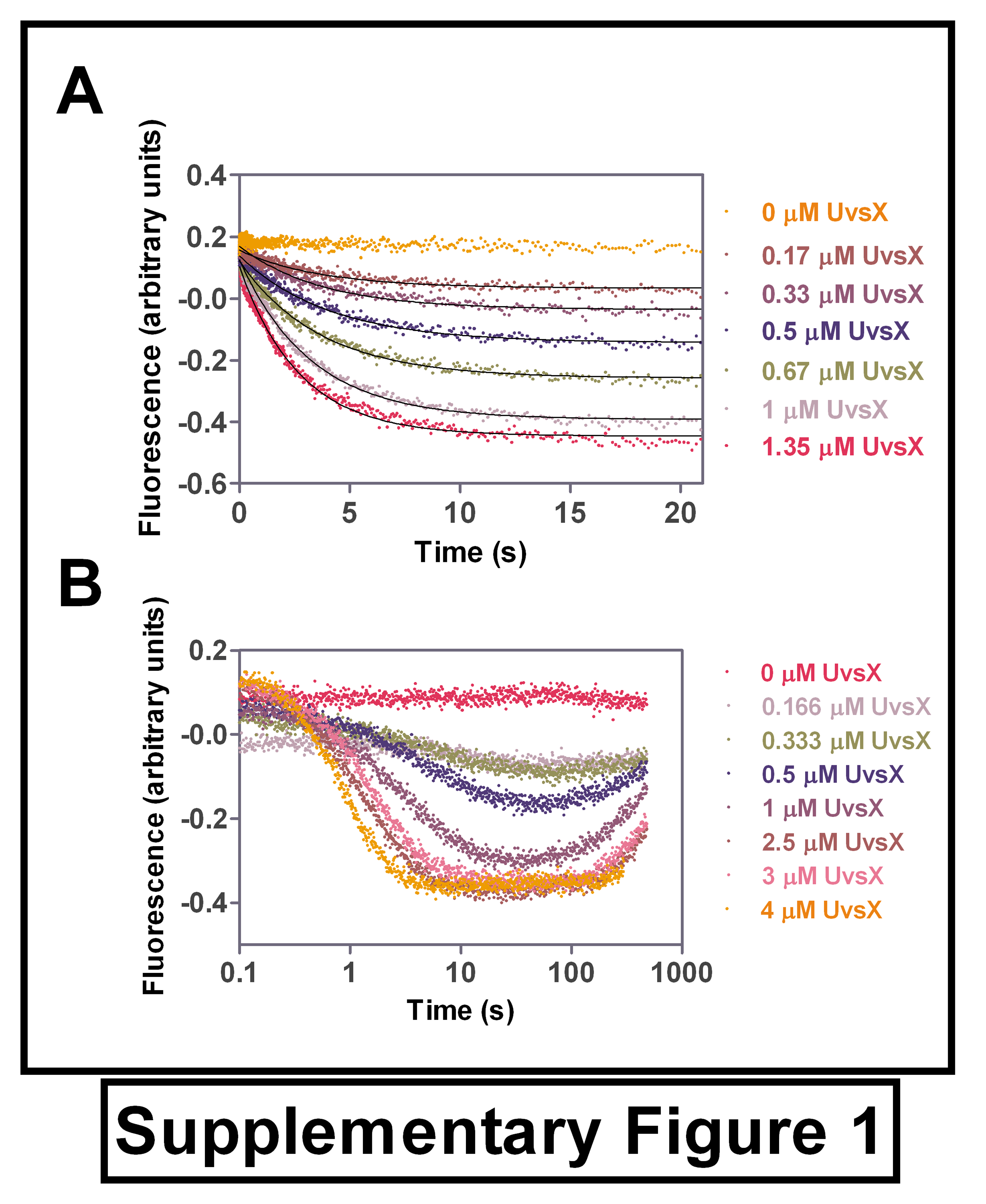

Supplement: Figure S1 — Typical traces of fluorescence quenching data used to measure dissociation constants for UvsX and labeled DNAs in the presence of nucleotide cofactors. UvsX hydrolyzes ATP in the presence of ssDNA. ATP hydrolysis is associated with the release of ssDNA. Thus the UvsX:ATP:ssDNA tripartite species is transient. In order to measure the affinity of UvsX for ssDNA in the presence of ATP rapid mixing techniques were used to observe ssDNA binding before ATP hydrolysis. We also used ATPγS, an ATP analogue which is hydrolyzed slowly. Reactions were initiated by the addition of UvsX (final concentrations indicated) to a mixture of 2 µM (nucleotides) ssDNA and (A) 2.5 mM ATP (final concentration) or (B) 900 µM ATPγS (final concentration). Reaction progress was monitored for up to 1000 s however only the first 30 s were used to determine the binding constants (Table 1). These data were fit to an exponential function to determine the total amplitude of quenching at each protein concentration. These amplitudes were then plotted as a function of UvsX concentration and these data were fit to Equation 1 to determine an apparent Kd. The similarity of the ATP and ATPγS fluorescence data as well as the similarity of the apparent Kd values obtained lead us to conclude that our techniques allowed us to measure the ATP bound form of the enzyme in the presence of ssDNA. (TIF) [file pone.0066654.s001.tif]

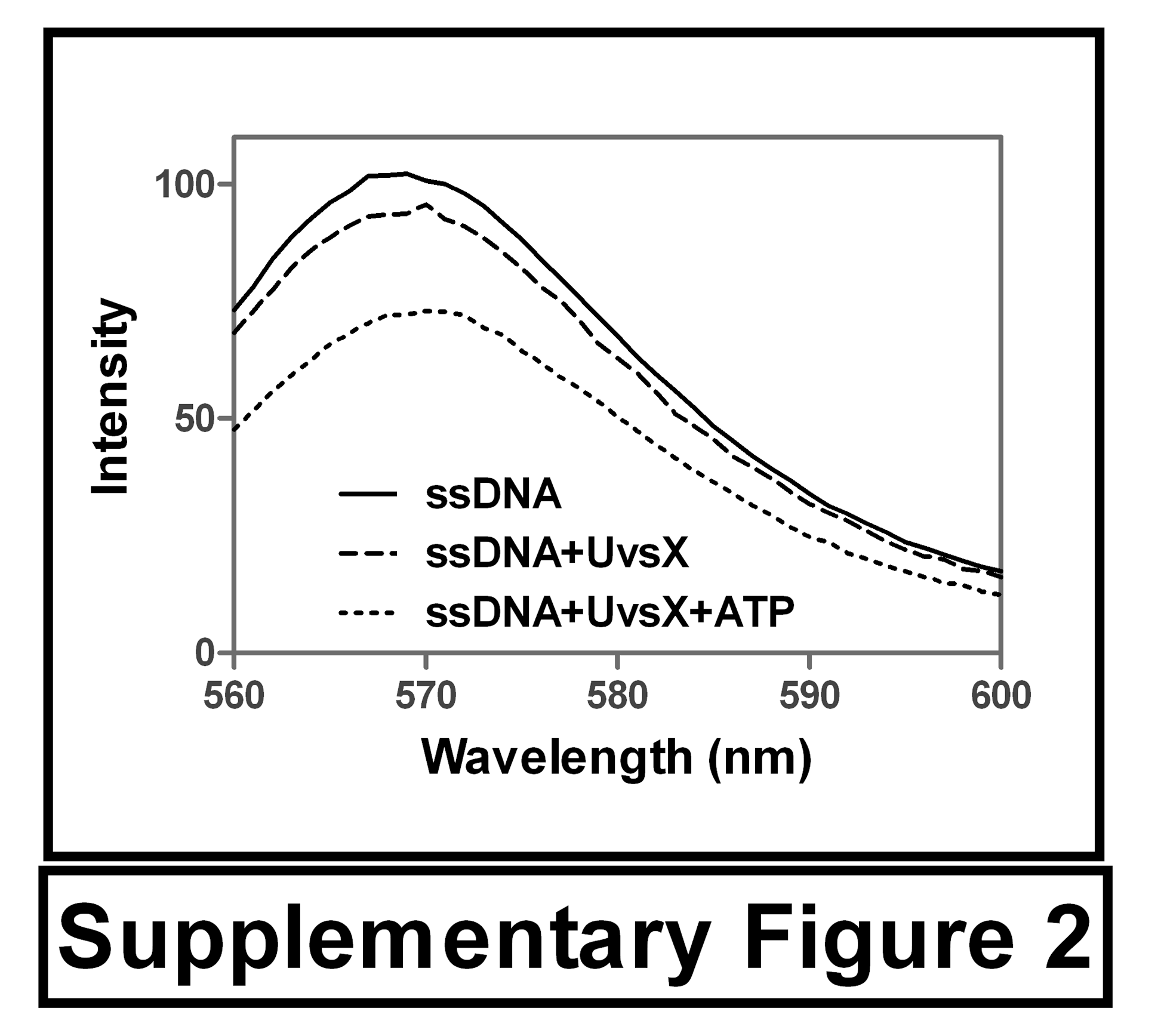

Supplement: Figure S2 — Effects of UvsX protein on fluorescence emission spectra of AlexaFluor 546-labeled oligonucleotide with alexafluor 546 differentially positioned. A single-stranded 25 mer oligonucleotide of the sequence dA25 with a 5′ C6 amino-modifier was covalenty labeled with AlexaFluor 546. The fluorescence emission from 560–600 nm with an excitation of 554 nm was recorded for 2 µM of this oligonucleotide alone (solid line), after the addition of 1.35 µM UvsX (dashed line) and after the addition of 3 mM ATP (dotted line). Reaction conditions were as described in the material and methods section for steady-state measurements. (TIF) [file pone.0066654.s002.tif]

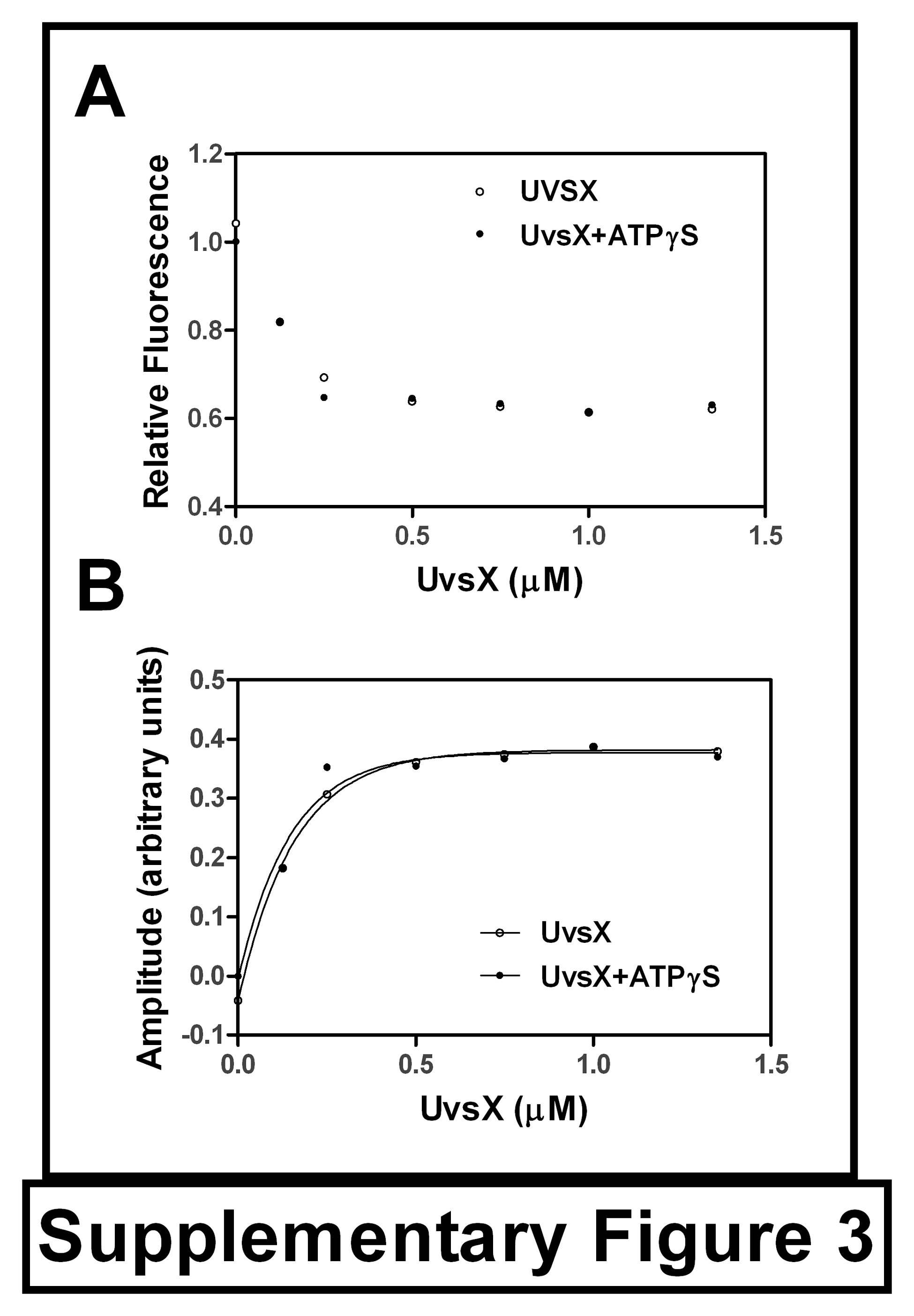

Supplement: Figure S3 — UvsX binding to 25 mer oliognucleotide 5′-dT22XT2-3′, in which X is amino-modifier C2 dT where the alexa 546 probe is covalently attached. 2uM ssDNA was added to various amounts of UvsX in the presence and absence of 900 µM ATPγS. The (A) fluorescence quenching relative to ssDNA alone and the (B) amplitude of fluorescence quenching was graphed as a function of UvsX concentration. (TIF) [file pone.0066654.s003.tif]

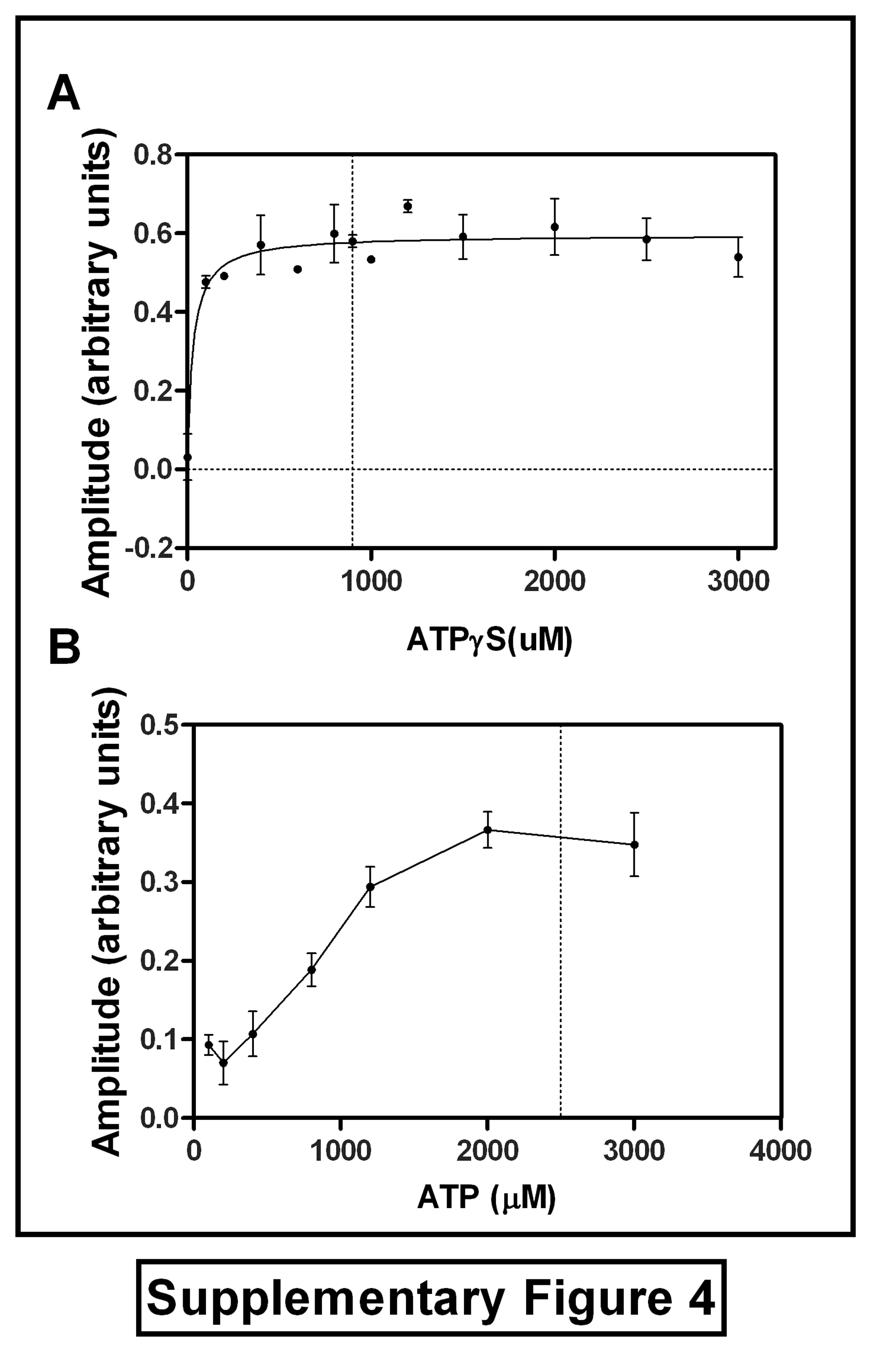

Supplement: Figure S4 — Titration of nucleotide cofactors to determine saturated binding conditions. A nucleotide cofactor is required for UvsX to binding to single-stranded oligo 1. ATP and ATPγS titrations were conducted to determine a saturating amount of ATP and ATPγS to be used in the ssDNA binding assays. Rapid mixing techniques with a SX.18 MV stopped-flow fluorometer (Applied Photophysics, Leatherhead, Surrey, UK) were used to measure binding of UvsX to ssDNA in the presence of the nucleotide cofactor before hydrolysis. 2 µM AlexaFluor 546 labeled oligo 1 and 1.35 µM UvsX were rapidly mixed with 0–3 mM ATP or ATPγS in a reaction buffer containing 20 mM Tris-HCl, pH 7.4, 50 mM NaCl, 3 mM MgCl2. The amplitude of fluorescence quenching was graphed as a function of ATP or ATPγS concentration. From these data 900 µM ATPγS and 2.5 mM ATP were chosen as saturating concentrations used to measure DNA binding by UvsX. (TIF) [file pone.0066654.s004.tif]
